# Supplementary material for: Knowledge, practices and perceptions of communities during a malaria larviciding randomized trial in the city of Yaoundé, Cameroon
Source: PLoS One. 2022 Nov 3;17(11):e0276500. doi: 10.1371/journal.pone.0276500 (PMC9632894; doi:10.1371/journal.pone.0276500)
Supplement: S1 File — (DOCX) [file pone.0276500.s002.docx]

**Interview Guide for Focus Groups and In-depth Interviews**

1. What actions do you think can be taken to effectively control malaria?

2. Are you aware of larval control activities taking place in your neighbourhood?

3. What do you think of this intervention?

4. In your opinion, what is the impression of the people in the neighbourhood about larviciding?

5. Could you tell us how we can improve the effectiveness of the larval control activities?

6. Do you think the application of larvicide will reduce the risk of malaria infection?

7. Did you noticed any change in the densities of mosquitoes biting you in the night?

8. Did you noticed any change in the number of malaria cases in your household?

9. Did you reduce the frequency of net use this year? Why or why not?

10. Can you tell us whether larviciding has helped reduce your annual spending on mosquito control and malaria treatment?

11. If given the tools would you be willing to conduct larval control in your neighbourhood?
