# Supplementary material for: Knowledge, practices and perceptions of communities during a malaria larviciding randomized trial in the city of Yaoundé, Cameroon
Source: PLoS One. 2022 Nov 3;17(11):e0276500. doi: 10.1371/journal.pone.0276500 (PMC9632894; doi:10.1371/journal.pone.0276500)
Supplement: S2 File — (DOCX) [file pone.0276500.s003.docx]

**
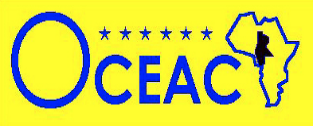
ORGANIZATION OF COORDINATION FOR THE FIGHT AGAINST**

Contacts :

-Dr N. Antonio : 699 53 86 56

- Dr A. Parfait : 699 836 111

**ENDEMIC DISEASES IN CENTRAL AFRICA**

**P.O.Box 15665, Yaoundé Cameroun**

**Tel : + 237 22 23 22 32**

**Fax : + 237 22 23 00 61**

**Web : http:// www.oceac.org**

***Household survey for population’s attitude towards malaria***

**Date**: ………………................… **Neighbourhood**………………………………………….**GPS**………………………………….

**Cluster** :............................................................................................... N° House.....................................................

**Respondent’s name** ......................................................................... Is he the head of the family? - Yes No

**Name and level of education of the head of the family** ................................................................................................................

**Head of the family’s job description** .......................................................................................................................

**Respondent’s level of education :** University - Secondary - Primary

**Water supply** : CamWater Water borehole - Well spring water

**Type of house** : - Cemented - Partially cemented - Wood - Mud blocks

**QUESTIONNAIRE**

**Question 1** : How many people live in the house ? ....................................................................................................

- Among the children, how many are under 5 years old ? . . . …………………………………………………………………………….

**Question 2 :** What do you think transmits malaria to humans ? …………………………………………………………………………………………………………………………………………………….

**Question 3 :** Do mosquitoes bite you in your house at night ? YES NO

**Question 4 :** If yes, what is (are) the protective measure(s) you use against mosquito bites?

- Mosquito nets - Mosquito screen on the windows - Repellents - Fan
- Spirals Insecticide spraying - Air conditioner -Others …………………………………….

**Question 5 :** if you use a mosquito net, when do you use it more?

- Rainy season - Dry season - Regularly Other ……….........................................................…

**Question 6:** How long have you had your mosquito net(s)?

- Less than 6 months - More than 6 months - More than a year - More than 2 years

**Question 7:** How did you get your mosquito net(s)?

-Distribution from Minsanté - Purchase - Donation - Other............................................................................

**Question 8 :** Do all the beds in the house have impregnated mosquito nets? - Yes - No

**Question 9:** How many mosquito nets do you have?.......................................................................................................

**Question 10:** Who are those that slept under a mosquito net last night?

- Everyone: - Parents: - Children : -Children <5 years old: -Visitors: Other............................

**Question 11:** Who regularly sleeps under a mosquito net at home?

- Everyone - Parents only - Children under 5 only -Other answer………………………….

**Question 12:** Why do you use the impregnated mosquito net?:

-To protect yourself from mosquito bites - Protect yourself from mosquito songs -To be able to sleep

-To avoid malaria Other...................................................................

**Question 13:** For those who do not frequently use the impregnated mosquito net, why don't they?

- Forgetfulness -Heat -No mosquitoes at home Other reasons.................................................................

**Question 14:** Is (are) your mosquito net(s):

- In good condition (without holes)? No.……… - Damaged (s) (presence of holes) No. ………

**Question 15**: : Do you know where mosquito larvae develop? - Yes - No

**Question 16**: Give examples of places where mosquito larvae develop ………………………………………… .....................................................................................................................................................................................................

**Question 17:** What do you do with stagnant pools of water around your house? …………………………………………………………………………………………………………………………………………………….

**Question 18**: What do you think are the symptoms (signs) of malaria?

…………………………………………………………………………………………………………………………….………..……………..…………………………………………………………………………………………………………………………………………………….

**Question 19**: What do you do when you suspect a case of malaria? (Assign the index 1,2 or 3 according to your preferences)

- Go to the hospital for consultation (1)
- Self-medication (you buy medication without a prescription) (2)
- Traditional medicine (3)
- .Order of preferences .......................................................................................................................................................

**Question 20: In case of self-medication or prescription by a doctor (nurse), where do you buy your medicines?**

- Pharmacy - Hospital/clinic Post (street)
- - Traditional healers -Others (specify)…………………………………….

**Question 21:** How much do you estimate your annual expenses :

- to fight against mosquitoes?......................................................................................................................................
- to treat malaria cases?....................................................................................................................................

**Question 22:** Do you agree that we have a light trap in your house at night (7 p.m. - 6 a.m.) to collect mosquitoes that enter and bite people inside the house as well as outside.

Yes No

Others.**.......................................................................................................................................................................................**
