# Supplementary material for: Knowledge, practices and perceptions of communities during a malaria larviciding randomized trial in the city of Yaoundé, Cameroon
Source: PLoS One. 2022 Nov 3;17(11):e0276500. doi: 10.1371/journal.pone.0276500 (PMC9632894; doi:10.1371/journal.pone.0276500)
Supplement: S3 File — (DOCX) [file pone.0276500.s004.docx]

**
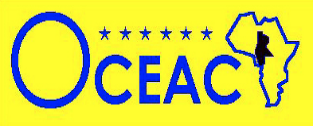

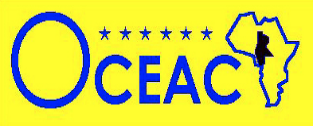
ORGANIZATION OF COORDINATION FOR THE FIGHT AGAINST**

**BP 15665, Yaoundé Cameroun**

**Tel : + 237 22 23 22 32**

**Fax : + 237 22 23 00 61**

**Web : http:// www.oceac.org**

Contacts :

-Dr N. Antonio : 699 53 86 56

- Dr A. Parfait : 699 836 111

Contacts :

-Dr N. Antonio : 699 53 86 56

- Dr A. Parfait : 699 836 111

**ENDEMIC DISEASES IN CENTRAL AFRICA**

**BP 15665, Yaoundé Cameroun**

**Tel : + 237 22 23 22 32**

**Fax : + 237 22 23 00 61**

**Web : http:// www.oceac.org**

***KAP household survey on malaria***

| **0.** **Socio-demographic data** | | |
| --- | --- | --- |
| 0.1 | Date |  |
| 0.2 | Investigator |  |
| 0.3 | Cluster |  |
| 0.4 | Participant’s code |  |
| 0.5 | Highest level of study of the head of household | 1 = University ;  2 = Secondary ;  4 = Primary ;  5 = Without level |
| 0.6 | Occupation of household head | 1= Mother ;  2= Father ; |
| 0.7 | Education level of respondent | 1 = University ;  2 = Secondary ;  4 = Primary ;  5 = Without level |
| 0.8 | Age of respondent |  |
| 0.9 | Sex of respondent | 1= Male 2= Female |
| 0.10 | Main source of drinking water | 1 = Camwater ; 2 = Water borehole ; 3 = Well ; 4 = Spring water |
| 0.11 | Type of house : | 1 = Cemented ; 2 = Mixed ; 3 = Wood ; 4 = Mud blocks |
| 0.12 | How many people are in the house? | A=Total number of people :  B=Children under 5 years old : |
| **1.** **ATTITUDE TOWARDS MALARIA** | | |
| 1.1 | What do you think transmits malaria to humans? |  |
| 1.2 | Can you name other mosquito-borne diseases? |  |
| 1.3 | Do you know where mosquitoes grow? |  |
| 1.4 | What do you do to protect yourself from mosquito bites? | A. Mosquito nets  B. Anti-mosquito screens on windows  C. Repellents  D. Fan  E. Spirals  F. Insecticide sprays  Others (please specify) |
| 1.5 | What do you think are the symptoms (signs) of malaria? |  |
| 1.6 | What do you do when you suspect a case of malaria? | 1= Hospital for consultation  2= ​​Self medication  3= traditional medicine.  **Order of preferences**……………………………………. |
| 1.7 | What are you doing to prevent malaria? |  |
| 1.8 | If you use a mosquito net, when do you use it more? | 1 = Rainy season; 2. = Dry season;  3 = Regularly  4= Others (specify) _________________ |
| 1.9 | Do all the beds in the house have mosquito nets? | 1= Yes 2= No |
| 1.10 | How many impregnated mosquito nets do you have? |  |
| 1.11 | How many people slept under a mosquito net last night out of how many in the house? | A. Total number ........................................  B. Children <5 years ..................................  C. Pregnant women.............................. |
| 1.12 | Why do you use the impregnated mosquito net? | 1 = To protect against mosquito bites  2 = Protect yourself from mosquito songs  3 = To be able to sleep;  4. = To avoid malaria |
| 1.13 | For those who do not use the treated net frequently, why don't they? | 1= Forgotten; 2= Heat; 3= No mosquitoes at home;  Others (please specify)............................... |
| 1.14 | What do you think can be done to reduce mosquito abundance? |  |
| **2. LARVAL CONTROL** | | |
| 2.1 | Do you know that mosquito control actions are carried out in your neighborhood to eliminate mosquitoes? | 1.Yes  2. No |
| 2.2 | If so, how did you first discover this activity? | 1.Talking with the treatment team  2. Seeing the treatment team at work  3. By being informed by the ward manager  4. By relatives |
| 2.3 | How do you think people in your neighborhood feel about this activity? |  |
| 2.4 | What have you seen during 2019 in terms of mosquito biting densities? | 1. Decrease ; 2. increase; 3. unchanged |
| 2.5 | What did you see during 2019 in terms of the number of malaria cases in your household? | 1. Decrease ; 2. increase; 3. unchanged |
| 2.6 | Did you reduce the frequency of use of nets during 2019? | 1. Yes ; 2. No   Why ? |
| 2.7 | In your opinion, what should be done to improve the effectiveness of larval control? |  |
| 2.8 | If ever you were given the tools, would you be ready to lead the fight against larvae in your neighborhood? | 1. Yes ; 2. No |
| 2.9 | During the year 2019, how much do you estimate your annual expenses to be :   1. For mosquito control.....................................   B. To treat malaria....................................... | |
