# Supplementary material for: Knowledge, practices and perceptions of communities during a malaria larviciding randomized trial in the city of Yaoundé, Cameroon
Source: PLoS One. 2022 Nov 3;17(11):e0276500. doi: 10.1371/journal.pone.0276500 (PMC9632894; doi:10.1371/journal.pone.0276500)
Supplement: Attachment — Submitted filename: Response to Reviewers.docx [file pone.0276500.s005.docx]

Organisation de Coordination pour

la lutte contre les Endémies en Afrique Centrale

Tel: 237 22 23 22 32

Fax: 237 22 23 00 61

**Dr ANTONIO-NKONDJIO Christophe**

Email: [antonio_nk@yahoo.fr](mailto:antonio_nk@yahoo.fr)

To

The Editor in chief

Plos One

Sir/Madam,

I will like to thank the reviewers and editor for their comments which substantially improved the quality of the manuscript. All requested changes were undertaken accordingly.

**Reviewer #1:**

**Specific comments**

**Comment 1:** The relationship between this manuscript and other related studies is not clear. In particular, the data included from 2017 appears to have been already published by Talipouo et al. 2019, but this is not made sufficiently clear in the manuscript.

**Answer 1:** Information included see end of the background section and information added in the legend of tables

**Comment 2:** The aims and questions/hypotheses to be tested in this paper need to be clearer. The objectives are described as to ‘evaluate people's knowledge and perception of the success of a pilot larviciding trial’. However, the results presented address several questions, including: the impact of the larvicidal intervention on LLIN use; the impact of the intervention on malaria/mosquito knowledge and practices; people’s perceptions of the intervention on mosquitoes and malaria cases; attitudes towards the intervention; and willingness to participate in larvicidal treatment. These are mostly presented as a comparison between intervention and non-intervention areas. If the aim of the paper was to assess how these metrics differ between the intervention and non-intervention areas, this should be stated clearly in the objectives and made clearer in the methodology. In the methodology make sure that it is clear which part of the study is being addressed by each part of the methods.

**Answer 2:** Corrected see in line 107-114. Further information was provided in the methodology see lines 197-215

**Comment 3:** A randomised control trial is mentioned in the title, but the processes of randomisation or assigning intervention and non-intervention areas are not explained. It appears that this control trial is described in Antonio-Nkondjio et al. 2021 (ref 30) but some detail is required here because it has important implications regarding whether the findings are robust, since it is plausible the findings could be explained by the method of selecting intervention and control areas.

**Answer 3:** We added in new section entitled selection of the intervention areas providing details on the way clusters were chosen and the study design, see lines 127-132

**Comment 4:** How were households selected for questionnaire administration in each cluster? Was this randomised? Were the same households visited in subsequent years? If so, this needs to be accounted for in the statistics. If not, this should be stated.

**Answer 4:** Corrected see in lines 143-146, the following information were added: “For each survey, a minimum of 50 households per cluster were randomly selected for the interview. Different households were interviewed each year. Parents (household head, spouse or an elder representative of the house) who consented to the study were interviewed”.

**Comment 5:** Binary measures e.g. ‘good practice’ and ‘good knowledge’ – how were these defined, how were the questionnaire data turned into a binary outcome?

**Answer 5:** Corrected see in line 154-165, the following information were added: “To assess the knowledge of respondents on malaria, the answers to four different questions including malaria signs and symptoms, mode of transmission, measures of prevention and knowledge of mosquito breeding habitats were combined. Participants providing correct answers to at least three of the questions were considered as having a good knowledge of malaria. Those who had less than 3 correct answers were considered as having poor knowledge of malaria. Concerning good practices in regard to malaria prevention and treatment, the answers to four different questions including sleeping under a treated bed nets regularly, going to hospital for malaria treatment, eliminating standing water bodies around houses and purchasing drugs in the pharmacy were assessed. Participants providing appropriate answers to at least three of the questions were considered as applying good practices while those with less than 3 corrects answers were considered having poor practices”.

**Comment 6:** How was the binary model constructed – what were the outcomes and explanatory variables? Was each assessed in a separate model?

**Answer 6:** Corrected see data analyses section, lines 206-209

**Comment 7:** How were the qualitative data analysed? What measures were taken to ensure the robustness of the analyses?

**Answer 7:** Information added see data analyses section lines 210-215

**Comment 8:** In the results, make sure it is clear what statistics have been used to generate the p-values indicated (for example line 220, line 231), and include test statistics

**Answer 8:** Corrected see results section

**Comment 9:** Check the wording for odds ratios – it should read ‘the odds of people having good knowledge was 2.64 times higher’ rather than people were 2.64 times more likely to be aware.

**Answer 9:** Changed throughout the text see lines 276-280

All requested changes were done accordingly and are highlighted in the main text. We look forward to hearing from you in due course.

Sincerely yours
